# Supplementary material for: Three-and-a-half million years of Tibetan Plateau vegetation dynamics in response to climate change
Source: Nat Ecol Evol. 2025 Jun 13;9(7):1153–67. doi: 10.1038/s41559-025-02743-2 (PMC12240865; doi:10.1038/s41559-025-02743-2)
Supplement: Supplementary file 1 — Supplementary Tables 2 and 4. [file 41559_2025_2743_MOESM1_ESM.pdf]

nature ecology & evolution

Supplementary information

https://doi.org/10.1038/s41559-025-02743-2

Three-and-a-half million years of Tibetan Plateau vegetation dynamics in response to climate change

In the format provided by the authors and unedited

Supplementary Table 2 | Vegetation types and their dominant taxa in the Zoige Basin and surrounding mountains[12, 13, 45]

Vegetation type	Elevation (m a.s.l)	Dominant species
Alpine sparse vegetation	4400-4600	*Saussurea* spp., *Rhodiola* spp., *Eriophyton wallichii*, *Androsace tapete*
Alpine shrubland and meadow	4000-4400	**Herb**: *Kobresia pygmaea*, *K. setschwanensis*, *Stipa capillacea*, *S. aliena*, *S. purpurea*, *Potentilla saundersiana*, *Anaphalis flavescens*, *Leontopodium junpeianum*, *Taraxacum dissectum*, *Saussurea* spp., *Bistorta vivipara*, *Bistorta macrophylla*, *Anemone* spp.<br><br>**Shrub**: *Rhododendron* spp., *Sibiraea angustata*, *Spiraea schneideriana*, *Salix sclerophylla*, *Dasiphora fruticosa*, *Hippophae tibetana*, *Juniperus pingii* var. *wilsonii*
Subalpine shrubland and meadow	3000-4000	**Herb**: *Kobresia setschwanensis*, *Elymus nutans*, *Elymus burchan-buddae*, *Festuca ovina*, *Poa pratensis*, *Anemone* spp., *Argentina anserina*, *Saussurea* spp., *Anaphalis flavescens*, *Leontopodium junpeianum*, *Bistorta vivipara*, *B. macrophylla*<br><br>**Shrub**: *Rhododendron* spp., *Sibiraea angustata*, *Spiraea schneideriana*, *Salix sclerophylla*, *Dasiphora fruticosa*, *Rubus* spp., *Caragana erinacea*, *Cotoneaster dielsianus*, *Quercus monimotricha*
Conifer forest	3000-4200	*Picea purpurea*, *P. likiangensis*, *P. wilsonii*, *P. asperata*, *P. brachytyla*, *Abies squamata*, *A. fargesii*, *A. fargesii* var. *faxoniana*, *A. georgei*, *Pinus densata*, *Juniperus tibetica*
Conifer and broadleaf mixed forest	2000-3000	*Tsuga chinensis*, *Pinus tabuliformis*, *Acer davidii*, *A. flabellatum*, *Betula utilis*, *B. platyphylla*, *B. albo-sinensis*, *Populus davidiana*, *Quercus baronii*
Evergreen and deciduous broadleaf mixed forest	1500-2000	*Lithocarpus cleistocarpus*, *Castanopsis platyacantha*, *Quercus glauca*, *Davidia involucrata*, *Betula insignis*, *Acer flabellatum*
Evergreen broadleaf forest	<1500	*Castanopsis platyacantha*, *Lithocarpus cleistocarpus*, *Quercus glauca*, *Schima sinensis*, *Pinus massoniana*, *Cunninghamia lanceolatas*

Supplementary Table 4 | Biome-taxon matrix used in the biome reconstruction. All terrestrial pollen taxa identified in the pollen assemblages from the Zoige Basin core are assigned to one or several biomes. Nineteen biome types are grouped into 5 mega-biomes.

**Mega-biome**	**Biome name**	**Biome abbreviation**	**Pollen taxa**
Forest	Cold deciduous forest	CLED	*Alnus*, Betula, Cupressaceae, Ericaceae, *Larix*, *Pinus*, *Salix*
	Cold evergreen needle-leaved forest	CLEG	*Abies*, *Alnus*, *Betula*, Cupressaceae, Ericaceae, *Larix*, *Picea*, *Pinus*, *Salix*
	Cold-temperate evergreen needle-leaved and mixed forest	CLMX	*Abie*, *Alnus*, *Betula*, Cupressaceae, Ericaceae, *Larix*, *Picea*, *Pinus*, *Salix, Tsuga*
	Cool evergreen needle-leaved forest	COEG	*Abies*, *Acer*, *Alnus*, *Betula*, *Corylus*, Cupressaceae, Ericaceae, Fabaceae, *Larix*, Oleaceae, *Picea*, *Pinus*, *Quercus*, Rosaceae, *Salix*, *Tilia*, *Tsuga, Ulmus*
	Cool mixed forest	COMX	*Abies*, *Acer*, *Alnus*, Anacardiaceae, Araliaceae, *Betula*, Caprifoliaceae, *Carpinus*, Celastraceae, *Celtis*, *Corylus*, Cupressaceae, Elaeagnaceae, Ericaceae, Euphorbiaceae, Fabaceae, *Hippophae*, *Ilex*, *Koelreuteria*, Lamiaceae, *Larix*, Malvaceae, Moraceae, Oleaceae, *Picea*, *Pinus*, *Quercus*, Rhamnaceae, Rosaceae, Rutaceae, *Salix*, Scrophulariaceae, Solanaceae, *Spiraea*, *Tilia*, *Tsuga*, *Ulmus*
	Temperate deciduous broad-leaved forest	TEDE	*Acer*, *Alnus*, Anacardiaceae, Araliaceae, *Betula*, Caprifoliaceae, *Carpinus*, *Carya*, *Castanea*, Celastraceae, *Celtis*, *Corylus*, Cupressaceae, Elaeagnaceae, Ericaceae, Euphorbiaceae, Fabaceae, *Fagus*, *Hippophae*, *Ilex*, *Juglans*, *Koelreuteria*, Lamiaceae, *Liquidambar*, Malvaceae, Moraceae, Oleaceae, *Pinus*, *Pterocarya*, *Quercus*, Rhamnaceae, Rosaceae, Rutaceae, *Salix*, Scrophulariaceae, Solanaceae, *Spiraea*, *Tilia*, *Ulmus*
	Warm-temperate evergreen broad-leaved and mixed forest	WTEM	*Acer*, *Alnus*, Anacardiaceae, Araliaceae, Boraginaceae, Caprifoliaceae, *Carpinus*, *Carya*, *Castanea*, *Cedrus*, Celastraceae, *Celtis*, Cupressaceae, Elaeagnaceae, Ericaceae, Euphorbiaceae, Fabaceae, *Fagus*, *Ilex*, *Juglans*, *Koelreuteria*, Linaceae, *Liquidambar*, Malvaceae, Moraceae, *Myrica*, Oleaceae, *Pinus*, *Podocarpus*, *Pterocarya*, *Quercus*, Rhamnaceae, Rosaceae, Rubiaceae, Rutaceae, *Salix*, Sapindaceae, Scrophulariaceae, Solanaceae, *Spiraea*, Thymelaeaceae, *Tilia*, *Tsuga*, Urticaceae
	Warm-temperate evergreen broad-leaved forest	WTEG	Anacardiaceae, Araliaceae, Boraginaceae, Caprifoliaceae, *Cedrus*, Celastraceae, Cupressaceae, Elaeagnaceae, Ericaceae, Euphorbiaceae, Fabaceae, *Ilex*, Moraceae, *Myrica*, Oleaceae, *Pinus*, *Podocarpus*, *Quercus*, Rhamnaceae, Rubiaceae, Rutaceae, Sapindaceae, Scrophulariaceae, Solanaceae, Thymelaeaceae, *Tsuga*
	Tropical semi-evergreen broad-leaved forest	TRSE	Anacardiaceae, Araliaceae, Boraginaceae, Caprifoliaceae, *Cedrus*, Celastraceae, Elaeagnaceae, Ericaceae, Euphorbiaceae, Fabaceae, *Ilex*, Malvaceae, Moraceae, *Myrica*, Oleaceae, *Podocarpus*, *Quercus*, Rhamnaceae, Rubiaceae, Rutaceae, Sapindaceae, Scrophulariaceae, Solanaceae, Thymelaeaceae, *Tsuga*, *Ulmus*, Vitaceae
	Tropical evergreen broad-leaved forest	TREG	Anacardiaceae, Araliaceae, Boraginaceae, *Cedrus*, Celastraceae, Convolvulaceae, Elaeagnaceae, Euphorbiaceae, Fabaceae, *Ilex*, Moraceae, *Myrica*, Oleaceae, *Podocarpus*, *Quercus*, Ranunculaceae, Rhamnaceae, Rubiaceae, Rutaceae, Sapindaceae, Scrophulariaceae, Solanaceae, Thymelaeaceae, *Tsuga*, *Ulmus*
	Tropical deciduous broad-leaved forest and woodland	TRDE	Anacardiaceae, Celastraceae, Euphorbiaceae, Fabaceae, Malvaceae, Oleaceae, Poaceae, Rubiaceae, Sapindaceae, *Ulmus*

Supplementary Table 4 | (continued)

Mega-biome	Biome name	Biome abbreviation	Pollen taxa
Shrubland	Erect dwarf-shrub tundra	DWAR	Cyperaceae, Ericaceae, *Hippophae*, Rosaceae, *Salix*
	Low and high shrub tundra	SHRU	Cyperaceae, Ericaceae, *Hippophae*, *Pinus*, Poaceae, Rosaceae, *Salix*, *Spiraea*
	Prostrate dwarf-shrub tundra	PROS	Apiaceae, *Artemisia*, *Aster*-type, other Asteraceae, *Betula*, Brassicaceae, Campanulaceae, Caryophyllaceae, Fabaceae, Gentianaceae, Geraniaceae, Lamiaceae, Liliaceae, Papaveraceae, Pedicularis, Polemonium, *Polygonum*, *Potentilla*, Primulaceae, Ranunculaceae, Rosaceae, *Salix*, *Saussurea*-type, Saxifragaceae, Scrophulariaceae, *Thalictrum*, *Taraxacum*-type
Meadow	Graminoid and forb tundra	DRYT	Apiaceae, *Artemisia*, *Aster*-type, other Asteraceae, Brassicaceae, Campanulaceae, Caryophyllaceae, Cyperaceae, Fabaceae, Gentianaceae, Geraniaceae, Lamiaceae, Liliaceae, Papaveraceae, *Pedicularis*, Poaceae, *Polemonium*, *Polygonum*, *Potentilla*, Primulaceae, Ranunculaceae, *Saussurea*-type, Saxifragaceae, Scrophulariaceae, *Thalictrum*, *Taraxacum*-type
	Cushion-forb tundra	CUSH	Apiaceae, *Artemisia*, *Aster*-type, other Asteraceae, Brassicaceae, Campanulaceae, Caryophyllaceae, Fabaceae, Gentianaceae, Geraniaceae, Lamiaceae, Liliaceae, Papaveraceae, Pedicularis, Polemonium, *Polygonum*, *Potentilla*, Primulaceae, Ranunculaceae, *Saussurea*-type, Saxifragaceae, Scrophulariaceae, *Thalictrum*, *Taraxacum*-type
Steppe	Temperate grassland	TEGR	Amaranthaceae, *Anthemis*-type, Apiaceae, *Artemisia*, *Aster*-type, other Asteraceae, Boraginaceae, Brassicaceae, Campanulaceae, Caryophyllaceae, Convolvulaceae, Cyperaceae, Ericaceae, Fabaceae, Gentianaceae, Geraniaceae, Lamiaceae, Liliaceae, Linaceae, Papaveraceae, Poaceae, *Polemonium*, *Polygonum*, *Potentilla*, Primulaceae, Rosaceae, Rutaceae, *Saussurea*-type, Saxifragaceae, Scrophulariaceae, Solanaceae, *Stellera*, *Thalictrum*, *Xanthium*, Zygophyllaceae, *Zygophyllum*
	Temperate xerophytic shrubland	TEXE	*Alhagi*, Amaranthaceae, *Anthemis*-type, Apiaceae, *Artemisia*, *Aster*-type, other Asteraceae, Boraginaceae, Brassicaceae, Campanulaceae, Caryophyllaceae, Convolvulaceae, Elaeagnaceae, Euphorbiaceae, Fabaceae, Gentianaceae, Geraniaceae, *Hippophae*, Lamiaceae, Liliaceae, Linaceae, *Nitraria*, Papaveraceae, *Polemonium*, *Polygonum*, *Potentilla*, Primulaceae, Rosaceae, Rutaceae, *Saussurea*-type, Saxifragaceae, Scrophulariaceae, Solanaceae, *Stellera*, Tamaricaceae, *Thalictrum*, *Xanthium*, Zygophyllaceae, *Zygophyllum*
Desert-steppe	Desert	DESE	*Alhagi*, Amaranthaceae, Elaeagnaceae, *Ephedra*, Euphorbiaceae, Fabaceae, *Hippophae*, *Nitraria*, Poaceae, Solanaceae, Tamaricaceae, Zygophyllaceae, *Zygophyllum*
