# Supplementary material for: Three-and-a-half million years of Tibetan Plateau vegetation dynamics in response to climate change
Source: Nat Ecol Evol. 2025 Jun 13;9(7):1153–67. doi: 10.1038/s41559-025-02743-2 (PMC12240865; doi:10.1038/s41559-025-02743-2)
Supplement: Supplementary file 2 — Reporting Summary [file 41559_2025_2743_MOESM2_ESM.pdf]

nature portfolio

Corresponding author(s): Yan Zhao

Last updated by author(s): Apr 30, 2025

Reporting Summary

Nature Portfolio wishes to improve the reproducibility of the work that we publish. This form provides structure for consistency and transparency in reporting. For further information on Nature Portfolio policies, see our Editorial Policies and the Editorial Policy Checklist.

Statistics

For all statistical analyses, confirm that the following items are present in the figure legend, table legend, main text, or Methods section.

n/a	Confirmed	
☐	☒	The exact sample size (*n*) for each experimental group/condition, given as a discrete number and unit of measurement
☒	☐	A statement on whether measurements were taken from distinct samples or whether the same sample was measured repeatedly
☐	☒	The statistical test(s) used AND whether they are one- or two-sided<br>*Only common tests should be described solely by name; describe more complex techniques in the Methods section.*
☒	☐	A description of all covariates tested
☒	☐	A description of any assumptions or corrections, such as tests of normality and adjustment for multiple comparisons
☐	☒	A full description of the statistical parameters including central tendency (e.g. means) or other basic estimates (e.g. regression coefficient) AND variation (e.g. standard deviation) or associated estimates of uncertainty (e.g. confidence intervals)
☒	☐	For null hypothesis testing, the test statistic (e.g. *F*, *t*, *r*) with confidence intervals, effect sizes, degrees of freedom and *P* value noted<br>*Give P values as exact values whenever suitable.*
☒	☐	For Bayesian analysis, information on the choice of priors and Markov chain Monte Carlo settings
☒	☐	For hierarchical and complex designs, identification of the appropriate level for tests and full reporting of outcomes
☒	☐	Estimates of effect sizes (e.g. Cohen's *d*, Pearson's *r*), indicating how they were calculated

Our web collection on statistics for biologists contains articles on many of the points above.

Software and code

Policy information about availability of computer code

Data collection: We obtain the samples from field coring and perform the laboratory analyses by ourselves to collect the data.

Data analysis: R 4.3.2, Matlab R2023a, Tilia 1.7.16, LPJ-GUESS 3.1 & 4.1, iLOVECLIM 1.2 and NorESM-L are used for data analyses in this study.

For manuscripts utilizing custom algorithms or software that are central to the research but not yet described in published literature, software must be made available to editors and reviewers. We strongly encourage code deposition in a community repository (e.g. GitHub). See the Nature Portfolio guidelines for submitting code & software for further information.

Data

Policy information about availability of data

All manuscripts must include a data availability statement. This statement should provide the following information, where applicable:

- Accession codes, unique identifiers, or web links for publicly available datasets
- A description of any restrictions on data availability
- For clinical datasets or third party data, please ensure that the statement adheres to our policy

The data are present in the paper and/or the Additional information. They are also available in the figshare (https://doi.org/10.6084/m9. figshare.27966036) database.

Research involving human participants, their data, or biological material

Policy information about studies with human participants or human data. See also policy information about sex, gender (identity/presentation), and sexual orientation and race, ethnicity and racism.

Reporting on sex and gender	Not applicable.
Reporting on race, ethnicity, or other socially relevant groupings	Not applicable.
Population characteristics	Not applicable.
Recruitment	Not applicable.
Ethics oversight	Not applicable.

Note that full information on the approval of the study protocol must also be provided in the manuscript.

Field-specific reporting

Please select the one below that is the best fit for your research. If you are not sure, read the appropriate sections before making your selection.

☐ Life sciences ☐ Behavioural & social sciences ☒ Ecological, evolutionary & environmental sciences

For a reference copy of the document with all sections, see nature.com/documents/nr-reporting-summary-flat.pdf

Ecological, evolutionary & environmental sciences study design

All studies must disclose on these points even when the disclosure is negative.

Study description	Biome changes on the eastern Tibetan Plateau for the past 3.5 million years are reconstructed based on fossil pollen records from the Zoige Basin, and the vegetation resilience and its response to climate changes are discussed.
Research sample	Two sediment cores from the Zoige Basin on the eastern Tibetan Plateau.
Sampling strategy	An average time resolution of ~700-year are retrieved for the fossil pollen records to capture long-term, orbital and millennial scale vegetation variability for the past 3.5 million years.
Data collection	Fossil pollen are extracted from the sediment samples of the drill core following standard procedures, and identified under optical microscope.
Timing and spatial scale	Mid-Pliocene to the present (3.5 million years), eastern Tibetan Plateau.
Data exclusions	No data are excluded from the analyses.
Reproducibility	The experiment and analysis procedures are well described in the manuscript.
Randomization	Randomization is not relevant to this study.
Blinding	Blinding is not relevant to this study.

Did the study involve field work? ☒ Yes ☐ No

Field work, collection and transport

Field conditions	Alpine meadow in the Zoige Basin, eastern Tibetan Plateau. Mean annual temperature and precipitation recorded at the Zoige meteorological station are 1.4°C and 650 mm, respectively.
Location	33°58′03.09″N, 102°20′09.76″E, 3442 m a.s.l.
Access & import/export	Field work were permitted by the Forestry and Grassland Bureau of Ruoergai (Zoige) County, and carried out in July-August 2019. All materials are collected in a responsible manner and in accordance with relevant permits and local laws.
Disturbance	No disturbance.

Reporting for specific materials, systems and methods

We require information from authors about some types of materials, experimental systems and methods used in many studies. Here, indicate whether each material, system or method listed is relevant to your study. If you are not sure if a list item applies to your research, read the appropriate section before selecting a response.

Materials & experimental systems

n/a	Involved in the study
☒	☐ Antibodies
☒	☐ Eukaryotic cell lines
☒	☐ Palaeontology and archaeology
☒	☐ Animals and other organisms
☒	☐ Clinical data
☒	☐ Dual use research of concern
☒	☐ Plants

Methods

n/a	Involved in the study
☒	☐ ChIP-seq
☒	☐ Flow cytometry
☒	☐ MRI-based neuroimaging

Plants

Seed stocks	Not applicable.
Novel plant genotypes	Not applicable.
Authentication	Not applicable.
